# Malaria vector control practices in an irrigated rice agro-ecosystem in central Kenya and implications for malaria control

**DOI:** 10.1186/1475-2875-7-146

**Published:** 2008-07-31

**Authors:** Peter N Ng'ang'a, Josephat Shililu, Gayathri Jayasinghe, Violet Kimani, Charity Kabutha, Lucy Kabuage, Ephantus Kabiru, John Githure, Clifford Mutero

**Affiliations:** 1 International Centre of Insect Physiology and Ecology (ICIPE), Nairobi, Kenya; 2 Jomo Kenyatta University of Agriculture and Technology, Nairobi Kenya; 3 International Water Management Institute (IWMI), Nairobi, Kenya; 4 Department of Community Health, University of Nairobi, Nairobi, Kenya; 5 College of Agriculture and Veterinary Science, University of Nairobi, Nairobi, Kenya; 6 Kenyatta University, Department of Pathology, School of Pure and Applied Sciences, Nairobi, Kenya; 7 International Water Management Institute (IWMI), Pretoria, South Africa

## Abstract

**Background:**

Malaria transmission in most agricultural ecosystems is complex and hence the need for developing a holistic malaria control strategy with adequate consideration of socio-economic factors driving transmission at community level. A cross-sectional household survey was conducted in an irrigated ecosystem with the aim of investigating vector control practices applied and factors affecting their application both at household and community level.

**Methods:**

Four villages representing the socio-economic, demographic and geographical diversity within the study area were purposefully selected. A total of 400 households were randomly sampled from the four study villages. Both semi-structured questionnaires and focus group discussions were used to gather both qualitative and quantitative data.

**Results:**

The results showed that malaria was perceived to be a major public health problem in the area and the role of the vector *Anopheles *mosquitoes in malaria transmission was generally recognized. More than 80% of respondents were aware of the major breeding sites of the vector. Reported personal protection methods applied to prevent mosquito bites included; use of treated bed nets (57%), untreated bed nets (35%), insecticide coils (21%), traditional methods such as burning of cow dung (8%), insecticide sprays (6%), and use of skin repellents (2%). However, 39% of respondents could not apply some of the known vector control methods due to unaffordability (50.5%), side effects (19.9%), perceived lack of effectiveness (16%), and lack of time to apply (2.6%). Lack of time was the main reason (56.3%) reported for non-application of environmental management practices, such as draining of stagnant water (77%) and clearing of vegetations along water canals (67%).

**Conclusion:**

The study provides relevant information necessary for the management, prevention and control of malaria in irrigated agro-ecosystems, where vectors of malaria are abundant and disease transmission is stable.

## Background

Malaria continues to be an important vector-borne disease and a leading cause of morbidity and mortality in Africa South of Sahara [1]. The disease is estimated to be responsible for 300–500 million clinical attacks globally and a minimum of between 1–2 million deaths annually [2]. It is a major threat to socio-economic development in the world and is also one of the major disease burdens in sub-Saharan Africa, where 15% of all disability life-years are lost to malaria [3]. The disease makes substantial demands on Africa's fragile health infrastructure, where the conventional treatment and control strategies have proved ineffective [4]. Pregnant women and children below the age of five years are at a higher risk of infection [5]. Current estimates indicate that at least one to three million children die each year in Africa alone. In Kenya, malaria is a major public health problem with its burden and transmission patterns varying across the country. Approximately 70% of the country is at risk of malaria infection and the disease accounts for 30% of all outpatients' attendance and 19% of all admissions in the health facilities [6]. There is considerable policy commitment by the Government of Kenya (GOK) to control malaria under the Division of Malaria Control. In order to achieve this, the government developed a 10-year National Malaria Strategy (NMS) plan in 2001 with an objective of reducing the level of malaria illness and death in Kenya by 30% and to sustain that improved level of control until 2010 [6]. However, malaria control in the country continues to experience many problems including the increasing spread of multi-drug resistant strains of *Plasmodium falciparum*, poverty, poor health infrastructure and ecosystem degradation.

Rice cultivation through irrigation has brought changes in the ecosystem which has affected the farmers' health in addition to creating habitats ideal for the breeding of vectors of diseases such as malaria and schistosomiasis [7]. This is in addition to changing the epidemiological pattern of malaria from seasonal to perennial, consequently raising the disease incidence in communities with little prior exposure or immunity [7,8]. Malaria transmission in most agricultural ecosystems is complex and involves the interactions of the host-vector-parasite triad, environment and the socio-economic factors in the community. Therefore, there is a need for developing a holistic malaria control interventions with adequate consideration of socio-economic factors which are equally important as biomedical, parasitological and entomological factors in determining infection and transmission of malaria in the community. A cross-sectional household survey was conducted in an irrigated ecosystem in Mwea Central Kenya with the aim of investigating vector control practices in the community and factors affecting their application both at household and community level.

## Methods

### Study site

**Figure 1:**
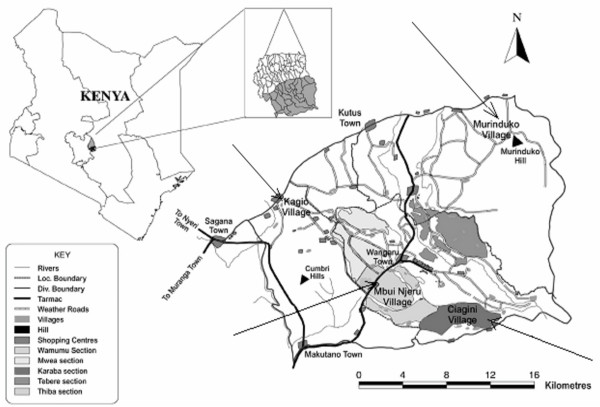
Map of Mwea Division with arrows indicating the four study villages.

The study was conducted in Mwea Division, Kirinyaga District in central Kenya, located approximately 100 km north-east of Nairobi, several kilometres south-east of Mt Kenya, at an altitude of about 1,159 m above sea level (Figure 1). The division has a population density of 246 persons per km^2 ^in a total area of 581 km^2^. The main economic activity is rice growing and horticultural farming with indigenous cattle kept mainly for beef and draught power. The area has two rainfall seasons with the long rains occurring from March to May and the short rains from October to November [9]. The annual rainfall varies from a maximum of 1,625 mm to a minimum of 356 mm, averaging 950 mm per annum. Average temperatures are in the range of 16 – 26.5°C and relative humidity varies from 52 – 67% [9-11]. This climatic condition provides suitable conditions for rapid development and survival of malaria vectors in the area. Due to rapid population growth and increasing demand for food, the hecterage under rice cultivation and numbers of cropping cycles continues to increase towards highland areas which were previously unknown for rice cultivation. This has led to increase in mosquito breeding habitats with possible alteration of the epidemiological pattern of malaria in a community with little prior exposure or immunity. Potential mosquito breeding habitats in the area include rice fields, feeder canals, temporary rain pools, run-offs, overflowing canals and puddles resulting from footprints of the workforce [9,11].

### Study design

The study was a cross-sectional household survey conducted from March to April 2005. Household census and village mapping was conducted in the four study villages in November and December 2004. This gave a framework for sample size selection where a standard sample of 100 households was randomly selected from each village. To create a framework of gender integration, at least 20% of the randomly selected sample comprised of female-headed households from each village.

### Data collection techniques

Interviews using structured questionnaires were conducted with a randomly selected individual heads of households or their spouse from the selected households in the four study villages. The questions focused on various sub-themes like, socio-demographic characteristic of the respondent, issues concerning knowledge and perceptions on malaria transmission and prevention as well as respondents' perceived effectiveness of available vector control practices. Questionnaires were prepared in English and verbally translated into the local language (Kikuyu) during interview time. Field assistants were selected from each study village and were centrally trained for two days on questionnaire administration techniques. Pre-testing was done in a non-study village and adjustments were made accordingly before final administration. Two focus group discussions (FGD) (one for men and the other for women) were held in each village in order to gather more descriptive information on people's knowledge, attitudes and vector control practices both at household and community level. The participants of the discussion consisted of an age-representative sample of men and women from each village. The questionnaire was administered after explaining purpose of the study and criteria used to select each respondent. Informed verbal and written consents were obtained from the participants and the local administration after going through the required study protocols. Confidentiality of information was maintained throughout the study period.

### Data management and analysis

Data was entered and processed using Statistical Package for Social Science (SPSS) version 11.5 and MS Excel. Association between dependent and independent variables were measured by use of Chi-square test.

## Results

### Socio-demographic characteristics of the respondents

In total, 368 households were successfully interviewed. They consisted of 127 (34.5%) males and 241 (65.5%) female respondents. Their ages ranged from 18 to 92 years, with median age of 39 years. Most of the respondents were protestants and formed 54.9% of the total sample followed by catholic (43.8%). Sixty-five percent (65%) of the respondents were married, 17.1% widowed, 8.2% single and 6.3% were separated at the time of interview. In terms of occupation, 74.5% of them were farmers, 7.3% in self business, 4.9% in formal employment and 13.3% in other minor occupations like casual labour. Forty percent (40%) of the respondents had only completed primary school education and 18.2% had dropped out at primary school level, 19.8% had informal education. There was a significant difference in the level of education of the respondents between the four study villages (χ^2 ^= 38.3; df 18; P = 0.004).

### Perceived causes of malaria

Mosquito bite was mentioned to be the main cause of malaria by 95% of the respondents. Other non-biological causes mentioned by some respondents were; long rains/being rained on (12.5%), which had a significant difference between the four villages (χ^2 ^= 24.336; df 3; P = 0.000), stagnant water (16%), dirty home surroundings (4.6%), wet and cold conditions (10.6%), eating raw of food/mangoes (5.2%) and taking of dirty or polluted water (4.1%). Significantly, more males (10.5%) compared to females (2.5%) believed that malaria could also be caused by eating raw food/mangoes (χ^2 ^= 10.19; df 1; P = 0.001). There was no significant difference in the responses between different levels of education of the respondents (Table 1).

**Table 1:** Perceived causes of malaria

**Perceived cause**	% Responses (n = 368)	χ^2 ^Test				
		
		Village	Age	Gender	Education	Occupation
Working in the sun	0.8	0.225	0.911	0.966	0.143	0.227
Long rains/Being rained on	12.5	0.000	0.908	0.709	0.093	0.061
Wet and cold condition	10.6	0.363	0.715	0.870	0.942	0.275
Working in rice paddies	3	0.451	0.174	0.930	0.083	0.255
Mosquito bite	94.6	0.154	0.000	0.914	0.850	0.973
Eating raw foods/mangoes	5.2	0.000	0.804	0.001	0.443	0.204
Evil spirit/Demons/Witchcraft	0.3	0.127	0.981	0.644	0.626	0.953
Taking dirty/Polluted water	4.1	0.081	0.009	0.648	0.210	0.850
From another person with malaria	0.8	0.479	1	0.687	0.805	0.382
Stagnant water	16	0.467	0.532	0.430	0.122	0.113
Dirty home surroundings/Environment	4.6	0.019	0.699	0.689	0.316	0.283
Don't know	1.1	0.532	0.000	0.687	0.135	0.846
Others	2.4	0.001	0.002	0.432	0.404	0.620

### Problems caused by mosquitoes

In total, 98% of the respondents said that mosquitoes caused trouble in one way or another to their households. Mosquitoes were reported to cause trouble by their nuisance bites by 75.3% of the respondents, while disease transmission was rated second by 64.4% of the respondents. The biting nuisance of the vector was elaborated during the FGDs, where the participants said the vectors bite through the clothes.

### Knowledge of vector-breeding habitats

Presumably as a result of their personal experience with the vector over a longer period of time, 83% of the respondents were aware of at least a major breeding habitat. Mosquito breeding sites mentioned were stagnant water found in swamps, rice paddies, water canals, hoof prints, tyre tracks and ponds. Other mentioned breeding places were: vegetation outside houses (51%), rubbish/pit latrines (16%), and animal pens (11%). There was no significant difference among the responses between the four villages (χ 9.938; df 6; P = 0.127) and also between gender of the respondents (χ^2 ^= 0.901; df 2; P = 6.37). A small proportion (2%) of the respondents was not aware of the major breeding sites of the vector in the area (Table 2). Nights (81%) and evenings (43%) were the most reported peak biting time for the mosquitoes in the study area.

**Table 2:** Knowledge of vector breeding habitats

**Breeding places**	% Responses n = 366	χ^2 ^Test				
		
		Village	Age	Gender	Education	Occupation
In stagnant water	83.1	0.233	0.782	0.320	0.054	0.104
In vegetations outside the house	50.5	0.030*	0.796	0.060	0.042*	0.191
In rice paddies	37.4	0.000*	0.945	0.016	0.351	0.158
In water canals	10.9	0.006*	0.345	0.228	0.140	0.235
In animal pens	10.7	0.001*	0.763	0.381	0.851	0.026*
Rubbish Pits/Latrines/Cess pits	16.1	0.000*	0.049	0.109	0.074	0.085
In dark places	23.2	0.006*	0.727	0.601	0.693	0.535
Don't know	1.6	0.673	0.009*	0.567	0.116	0.641
Others	3.8	0.378	0.056	0.922	0.758	0.699

*Significant

### Malaria vector control practices

#### Personal protection methods applied

Seventy-five per cent (75%) of the respondents reported to own at least a bed net during the time of interview. Of these, 62% were insecticide-treated and the remaining 38% were untreated. Use of treated bed nets varied significantly between different socio-demographic profiles, including gender (χ^2 ^= 4.254; df 1; P = 0.039), level of education (χ^2 ^= 33.622; df 6; P = 0.000), marital status (χ^2 ^= 19.593; df 3; P = 0.000) and occupation (χ^2 ^= 7.955; df 3; P = 0.047). Use of insecticide sprays was reported by 7.1% of respondents. Among those who reported use of the method during interview time included, 4.4% of the farmers, 3.7% of business people, and 27.8% of the respondents in formal employment. There was a significant difference in use of insecticide sprays among different occupational groups (χ^2 ^= 23.023; df 3; P = 0.000) and also between the four villages (χ^2 ^= 7.159; df 3; P = 0.067). In total, screening of windows and doors was reported to be in use by 3% of the respondents who happened to be from two villages, Ciagi-ini and Mbui-njeru. Seventeen percent (17%) of respondents acknowledged lighting fire and mosquito coils for personal protection against mosquito bites at night. This consisted of 17.2% of the farmers, 25.9% of the business people, 33.3% of those with formal employment and 8.2% of respondents with other minor occupations like casual labours. Application of fires and mosquito coils among different occupational groups showed significance variation between the four study villages (χ^2 ^= 45.276; df 3; P = 0.000). Traditional methods (burning of cow dung and local herbs) were used by 7.9% of the respondents. They were reported in small percentages in the study area but with a significant variation in their use between the four villages (Table 3).

**Table 3:** Personal protection methods applied at household level

**Method**	% Applied	χ^2 ^Test					
		
		Village	Age	Gender	Education	Occupation	Religion
Untreated mosquito net	34.9	0.000	0.849	0.502	0.552	0.090	0.162
Treated Mosquito net	57.2	0.002	0.077	0.037	0.000	0.090	0.203
Insecticide spray	5.9	0.067	0.846	0.363	0.089	0.000	0.548
Preventive medicine	11.5	0.291	0.631	0.091	0.610	0.148	0.716
Screen windows & doors	3.3	0.017	0.889	0.296	0.318	0.701	0.895
Light fire/Coils	21.1	0.000	0.866	0.372	0.278	0.076	0.318
Skin repellents	1.6	0.600	0.895	0.795	0.604	0.488	0.947
Traditional methods	7.9	0.018	0.651	0.301	0.854	0.368	0.093

#### Reasons for non-use of available personal protection methods

Despite malaria being reported as one of the most frequently occurring diseases in the area, most respondents could not apply regularly some of the available personal protection methods. Unaffordability (67.7%) was the main reason cited for regularly non-use. Other reasons given for regular non-use were; side effects (26.6%), lack of effectiveness (21.5%), low mosquito density and lack of application know how with 6.5% each, (n = 368). Reasons cited for non-use varied depending on the type of the practice. Practices, which most respondents said they could not afford to apply regularly were; use of treated nets (91.7%), use of; insecticide sprays (50%) and use of skin repellents (36%). The socio-economic issue was clarified during FGDs. It was emphasized that household had other daily responsibilities like paying school fees and feeding the family in addition to protecting the household against other common diseases. Other reasons cited included lack of effectiveness of some practices like untreated nets (59.7%) and side effects of some methods like use of fire/coils (50%) and traditional methods like burning local herbs and cow dung (78.6%). Traditional methods like use of cow dung and burning of local herbs were reported to have side effects ranging from respiratory/breathing problems, eye irritation, coughing, colds and flu. Cow dung was said to releases too much smokes in the house, leaving everything in the house smelling smoke.

### Environmental management practices

**Figure 2:**
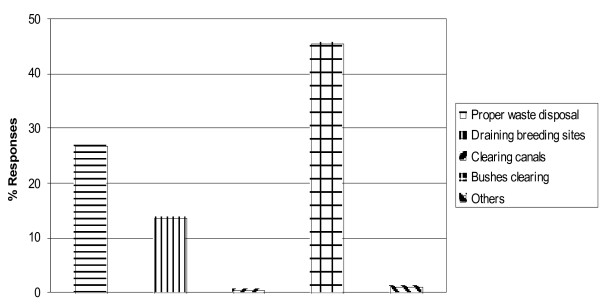
**Environmental management practices at household level**.

Both household and community level environmental managements practices were reported. Among the reported practices at household level included; clearing household refuse/proper waste disposal (26.9%), draining/leveling of breeding sites around houses (13.6%), clearing of vegetations in canals (0.5%) and clearing of bushes and vegetations around houses (45.7%; Fig 2). Community level environmental management practices reported were; levelling and draining areas of stagnant water (4.3%), clearing vegetations in water canals (0.3%) and destruction of discarded water receptacles in the village (1.1%).

#### Reasons for non-application of environmental management

Lack of time was the most cited response for regular non-application of environmental management practices both at household and community level (56.3%). Other responses cited for non-use were the presence of low mosquito densities (13.4%) and lack of effectiveness (9%). Methods that most users said they could not apply regularly at household level due to lack of time were levelling/draining breeding sites (77.4%) and clearing of vegetations in canals (66.7%). Similarly, at community level, levelling/draining stagnant water pools (50%) and clearing of vegetations in water canals (66.7%) were the most cited measures which majority of respondents said they could not get time to regularly apply. Low mosquito density was the sole reason given for not regularly destroying discarded receptacles by the applicants.

## Discussion

Results from this cross-sectional study showed that mosquitoes were recognised to be important vectors of malaria in Mwea. It was noted that 95% of the respondents recognized the role of mosquitoes in malaria transmission. However, most respondents (74%) reported that the vector caused most trouble to their family by its biting nuisance. This was confirmed during focus group discussions (FGDs), when the participants reported that it was difficult to sleep comfortably at night without a bednet. Similar observations have been made in other studies in different parts of the world where the nuisance role of the mosquito have been cited as the main reason making most households spend money on personal protection measures [13,14]. Such an attitude of linking the vector with its biting nuisance among the residents could negatively influence the choice, use and sustainability of an intervention measure aimed at controlling malaria. This is because, the intervention could be perceived to be geared towards eliminating mosquitoes as a biting nuisance, but not necessarily aimed at reducing the suffering resulting from malaria morbidity and mortality.

Apparently, as result of their long personal experience with the nuisance biting behaviour of mosquitoes most respondents in Mwea were familiar with the vector ecology and behaviour. The role played by stagnant water in mosquito breeding was recognized by most respondents, however, some respondents did not know where the vector breeds while others mentioned sites which were not specifically related to water (e.g. in vegetations outside the house) and it was not clear whether these were thought to be the breeding or hiding places. A good knowledge and understanding of the breeding habitats of the malaria vector among the residents forms an important background in decision-making process [15]. Both traditional and orthodox methods of controlling mosquitoes were used in the study area. However, environmental management practices were not in high use both at household and community level. Overall, only 7% of the respondents participated in environmental management at community level. This low percentage of involvement depicted the level of active community participation and social relationship in organized community efforts in malaria control practices in Mwea. This was justified during one of the FGDs, where the participants contrasted current situation with what used to be some years back when the public health officers and local administration used to enforce measures of environmental management, sanitation and overall health education, which were aimed at general hygiene. It was a clear indication that people cooperated with such practices because they were enforced by the administration and feared legal action. They perceived the enforcement as a government policy that had to be obeyed without any question. Therefore, without legal enforcement, community were unwilling to participate. However, if well formulated and implemented, community participation in vector control can have a significant and sustainable impact on vector density. This is rarely achieved because most interventions are vertically structured indicating that community members are not involved in the design and implementation stages. Communities can easily be motivated by linking the programmes to income generating activities, which are socially, culturally and economically appropriate [16,18].

The role played by housing design and screening of windows, doors and eaves in mitigating malaria transmission was less mentioned. In most tropical rural areas, where malaria is endemic, Mwea included, housing is epitomized by flimsy, open-walled structures, overcrowding, poor ventilation, open eaves coupled with little protection from insects that spread diseases like malaria. The unscreened windows and eaves provide easy access for the vectors of human malaria [19-21]. Improving the design of these traditional dwellings could significantly reduce the number of infective bites, resulting into reduced disease transmission in the study area. During the Focus Group Discussions, traditional methods were said to be partially effective in driving away mosquitoes in all the study villages, participants said that when the smoke went off, the mosquitoes 'rushed on again'. Though the efficacy of traditional methods has not been elucidated, their use for personal protection can be a valuable investment. They can be applied in situations where other protective measures are impractical particularly for people who work outdoors at night when the insect-biting rate is high, and when people sleep outside to flood rice paddies, water horticultural crops or guard rice crops during harvesting time. They can also be used indoors in combination with other personal protection methods especially in the early evening before people retire to bed or the early morning before sunrise when people are not protected by other methods like bednets [22-24].

For any vector control intervention to be sustainable in the community, it has to be technically, economically and socially sound [24]. This implies that it must be effective, affordable, acceptable and compatible with the local customs attitudes and beliefs. Generally, people opt to choose actions that are less expensive, save, simple and easier to administer [25]. In Mwea, unaffordability (socio-economic status) was the main reason for not using regularly some of the available vector control interventions like treated mosquito net, insecticide spray and lighting of coils in Mwea. During FGDs in one of the non-irrigated village (Murinduko), which was relatively poorer socio-economically, it was revealed that insecticide-treated bed nets were unaffordable and their ownership and use was viewed to be a preserve for the rich. Large-scale rice cultivation provides farmers with disposable incomes which they invest in personal protection measures such as insecticide-treated bed nets, resulting into reduction in malaria incidences as a result of better protection against anthropophilic mosquitoes [26]. In most cases, what may appear affordable to the outside through socio-economic based measurement (e.g. household income, house design, among others) might not be affordable to many households. During the focus group discussion, it was recognized that households have many competing needs (e.g. food and education) and tradeoffs. Therefore, households without sustainable money upfront may result to other perceived less expensive traditional methods like burning local herbs, cow dung, and wood fires, which actually may not be effective in the long run.

Another reason given for regular non-use was lack of effectiveness. Contrary to epidemiological or scientific indicators of effectiveness as described by Lengeler and Snow [27], local people mostly determines the effectiveness of a vector control intervention by its immediate or noticeable potential in either reducing adult mosquito population, stopping the nuisance biting or reducing the breeding habitats. Failure to satisfy the above conditions, the intervention would be deemed ineffective and this can make community support either to wane or develop negative perception. This implies that for vector control interventions to be sustainable they must be effective, affordable, acceptable and compatible with the local customs attitudes and beliefs. For Mwea people, untreated bed nets were ineffective in reducing the nuisance biting. This was the main reason given for their reported non-use by most respondents. Comparatively, treated bed nets were said to offer better protection against mosquito bites in all the four study villages. This was through its killing and excito-repellent effects of pyrethroids which cause the mosquitoes to leave rooms for the outdoors, resulting to observed reduction in indoor biting [28,29]. Traditional methods and environmental management practices were said to be partially effective in reducing man-vector contact. Practically, environmental management practices are normally not very effective by themselves and they need to be integrated with other control measures. This is because they do not have immediate effect in reducing the number of biting vectors and may take several days or weeks before reduction in their numbers can be achieved or appreciated. They have significant impact only if they cover relatively high proportions of breeding sites within vector flight range and large proportion of community members actively participate. Besides being not well known, greater part of the respondents said that they never had time to apply most of the environmental management practice both at household and community level. In conclusion, this study has provided a number of relevant information, which needs to be considered and integrated into the design and implementation of malaria control strategies. This should ensures that interventions are sustainable, culturally appropriate and economically feasible both at individual and community level.

## Competing interests

The authors declare that they have no competing interests.

## Authors' contributions

PN conducted the field work. JS and GJ provided scientific guidance in data collection, data analysis and manuscript preparation. VK, LK, EK, JG and CM provided guidance in data collection and supervision of the project. All authors read and approved the final manuscript.
